# Supplementary material for: Content-rich biological network constructed by mining PubMed abstracts
Source: BMC Bioinformatics. 2004 Oct 8;5:147. doi: 10.1186/1471-2105-5-147 (PMC528731; doi:10.1186/1471-2105-5-147)
Supplement: Additional File 2 — The original results of the above study (non-essential files are deleted to keep the file size under the limit set by BMC bioinformatics). [file 1471-2105-5-147-S2.bz2 › chilibotAdditionalFile2/dip10/760ID10756017E18146/html/DNAB_B.html]

 


 **DNAB** and **B** 
  
Found 69 abstracts in PubMed, retrieved 10.  
 

 What does Google say? 
 PDF only 
| .edu only 

---

**Interactive relationship** (e.g. stimulation, inhibition, etc)

**Neutral relationship**- In this paper we provide evidence for a functional interaction between the primosomal protein DnaI from  **B** .subtilis and the main hexameric replicative helicase < **b** > DnaB  **b** > from Bacillus stearothermophilus.  Ref: 11842108 Nucleic Acids Res, 2002
- DnaD alone and the DnaD < **b** > DnaB  **b** > pair interact specifically with PriA of  **B** . subtilis on several DNA substrates.  Ref: 11585815 J Biol Chem, 2001
- These observations confirm that < **b** > DnaB  **b** >, DnaD and DnaI are primosomal proteins in  **B** . subtilis.  Ref: 11679082 Mol Microbiol, 2001
- Other selected mutations that map to regions carrying Walker A and  **B**  boxes are expected to be defective in ATP binding, a required step in < **b** > DnaB  **b** > DnaC complex formation.  Ref: 11333269 J Biol Chem, 2001
- Here, we describe < **b** > dnaB  **b** > mutations that suppress the phenotypes of  **B** . subtilis priA mutants.  Ref: 11679082 Mol Microbiol, 2001

**Non-interactive relationship** (e.g. studied together, co-existance, homology, etc.)

- We have proposed previously that three proteins involved in the initiation of replication at oriC in  **B** . subtilis, < **b** > DnaB  **b** >, DnaD and DnaI, are components of the PriA primosome in this bacterium.  Ref: 11679082 Mol Microbiol, 2001
